# Expansion of Known Rhodobacter capsulatus Bacteriophage Diversity with 24 Additional Genomes

**DOI:** 10.1128/mra.00879-22

**Published:** 2022-11-03

**Authors:** Amanda Wilson, Joselyn Molinar, Josephine Aldrich, Gnandeep Chintala, Katy Smit, Kai Miller, David W. Bollivar, Richard M. Alvey

**Affiliations:** a Department of Biology, Illinois Wesleyan University, Bloomington, Illinois, USA; Portland State University

## Abstract

We report the genome sequences of 24 newly discovered bacteriophages that infect Rhodobacter capsulatus, a model for photosynthesis and horizontal gene transfer studies. All have substantial relatedness to previously reported siphovirus bacteriophages. Most are categorized in known clusters (RcB, RcC, RcD, and RcF), with one forming a new cluster, RcG.

## ANNOUNCEMENT

Rhodobacter capsulatus, a freshwater alphaproteobacterium, was the first microorganism found to perform horizontal gene transfer with a bacteriophage-like gene transfer agent (RcGTA) (1). Recent studies found that many bacteriophages that infect this host share genes with RcGTA (2–6). Prior to this announcement, 29 R. capsulatus phage genomes were organized into six distinct clusters (RcA to RcF), with four remaining as singletons (2). To advance our understanding of these phages and RcGTA, we have expanded this collection with 24 genomes from isolates collected throughout the state of Illinois.

Phages were discovered using previously described enrichment protocols, where 10-mL environmentally collected water samples were combined with 10 mL of fresh yeast extract-peptone medium and 10 mL of R. capsulatus strain YW1 culture grown to an optical density at 600 nm (OD_600_) of ~1.0 (2). After 3 to 5 of days shaking at 30°C, samples were filtered through 0.22-μm filters and plated with a 0.4% top agar overlay embedded with R. capsulatus cells. Plates were examined for plaques after a 1- to 3-day 30°C incubation. After 2 to 4 single-plaque isolations, DNA was extracted from high-titer (>5.0 × 10^7^ PFU/mL) lysates with the Wizard DNA prep kit (Promega), and Illumina sequencing was conducted using either the MiSeq or NextSeq 2000 platform (Table 1). Libraries were prepared using the TruSeq Nano DNA library kit (Illumina), with Covaris shearing. Resulting 150-bp single-end reads were assembled using PATRICBRC.org (7) or Newbler v.2.9 and assessed for quality and completeness using Consed v.29 (8). Annotations used DNAMaster v. 5.23.6 (9) and Pecaan v. 20210526 (https://blog.kbrinsgd.org/), with GeneMark v. 3.25 (10), GLIMMER v.3.02 (11), NCBI BLAST v.2.9.0 (12), tRNAscan-SE v.2.0 (13), ARAGORN v.1.2.38 (14), HHpred v.3.2.0 (15), and Phamerator v.326 (16), all with default parameters.

**TABLE 1:** Genometrics of 24 additional R. capsulatus bacteriophages

Phage name	GPS coordinates (°)	Collection date (mo/day/yr)	Sequencing platform	Cluster	Genome length (bp)	G+C content (%)	Coverage (no. of reads)	No. of CDSs^*a*^	GenBank accession no.	SRA accession no.
RcMotherGoose	40.533539, −88.994855	5/19/2022	Illumina NextSeq 2000	RcB	44,429	55.2	7,664	63	OP009282	SRX17116970
RcKai	40.546383, −88.956529	9/15/2020	Illumina NextSeq 2000	RcC	41,645	64.2	3,524	68	OP009272	SRX17116951
RcKvothe	40.496051, −88.990428	10/10/2019	Illumina MiSeq	RcC	41,447	64.2	1,712	67	OP009275	SRX17116967
RcMamaDuck	40.468213, −88.970813	5/19/2022	Illumina NextSeq 2000	RcC	41,838	64	7,202	69	OP009278	SRX17116950
RcMeacham	41.962013, −88.072160	9/28/2019	Illumina MiSeq	RcC	41,198	64.2	1,518	67	OP009280	SRX17116968
RcSwan	40.503560, −88.926892	9/17/2021	Illumina NextSeq 2000	RcC	41,417	64.2	7691	68	OP009286	SRX17116962
RcAqua	40.503066, −88.930833	9/17/2021	Illumina NextSeq 2000	RcD	66,472	60.2	124	95	OP009265	SRX17116953
RcAquaphina	40.326284, −88.570188	9/19/2020	Illumina NextSeq 2000	RcD	68,972	59.9	7,459	102	OP009266	SRX17116964
RcBigEagle	40.531708, −88.932522	5/23/2022	Illumina NextSeq 2000	RcD	68,138	60.2	5,069	100	OP009267	SRX17116954
RcCWillis	40.495769, −88.989552	9/14/2021	Illumina NextSeq 2000	RcD	68,804	61.2	5,671	97	OP009268	SRX17116973
RcDora	40.494485, −89.012498	9/16/2021	Illumina NextSeq 2000	RcD	67,639	60.2	7,620	100	OP009269	SRX17116965
RcExplorer	40.459407, −88.948256	9/11/2021	Illumina NextSeq 2000	RcD	68,400	60.1	6,986	102	OP009270	SRX17116959
RcJoli	39.867163, −89.621677	8/9/2019	Illumina MiSeq	RcD	68,547	60.1	1,233	101	OP009271	SRX17116958
RcKeef	40.496629, −88.991761	9/1/2021	Illumina NextSeq 2000	RcD	68,260	60	7,366	101	OP009273	SRX17116963
RcLkye	40.494807, −88.987354	9/13/2021	Illumina NextSeq 2000	RcD	68,442	60	6,818	101	OP009276	SRX17116961
RcMaeve	41.630898, −88.293465	10/1/2016	Illumina MiSeq	RcD	65,779	60.2	1,775	94	OP009277	SRX17116966
RcMcLean	40.364950, −88.964090	9/25/2021	Illumina NextSeq 2000	RcD	67,427	60.2	6,477	94	OP009279	SRX17116957
RcPacific	40.198975, −88.393187	9/12/2017	Illumina MiSeq	RcD	67,036	60.2	376	98	OP009283	SRX17116952
RcPeripeteia	40.494456, −89.012508	10/13/2019	Illumina MiSeq	RcD	66,287	60.1	331	96	OP009284	SRX17116960
RcWata	41.873038, −87.826485	9/5/2016	Illumina MiSeq	RcD	67,804	60.2	2,566	97	OP009287	SRX17116955
RcWhiteOak	40.495642, −89.012511	9/22/2020	Illumina NextSeq 2000	RcD	68,141	60.4	7,353	101	OP009288	SRX17116956
RcKickapoo	40.364950, −88.964090	9/25/2021	Illumina NextSeq 2000	RcF	94,139	57.7	4,337	136	OP009274	SRX17116971
RcMenchie	41.928985, −87.844700	8/22/2017	Illumina NextSeq 2000	RcF	94,440	57.8	3,674	141	OP009281	SRX17116972
RcRudolph	40.468213, −88.970813	6/1/2021	Illumina NextSeq 2000	RcG	64,892	65.3	4,032	85	OP009285	SRX17116969

a CDSs, coding sequences.

**FIG 1:**
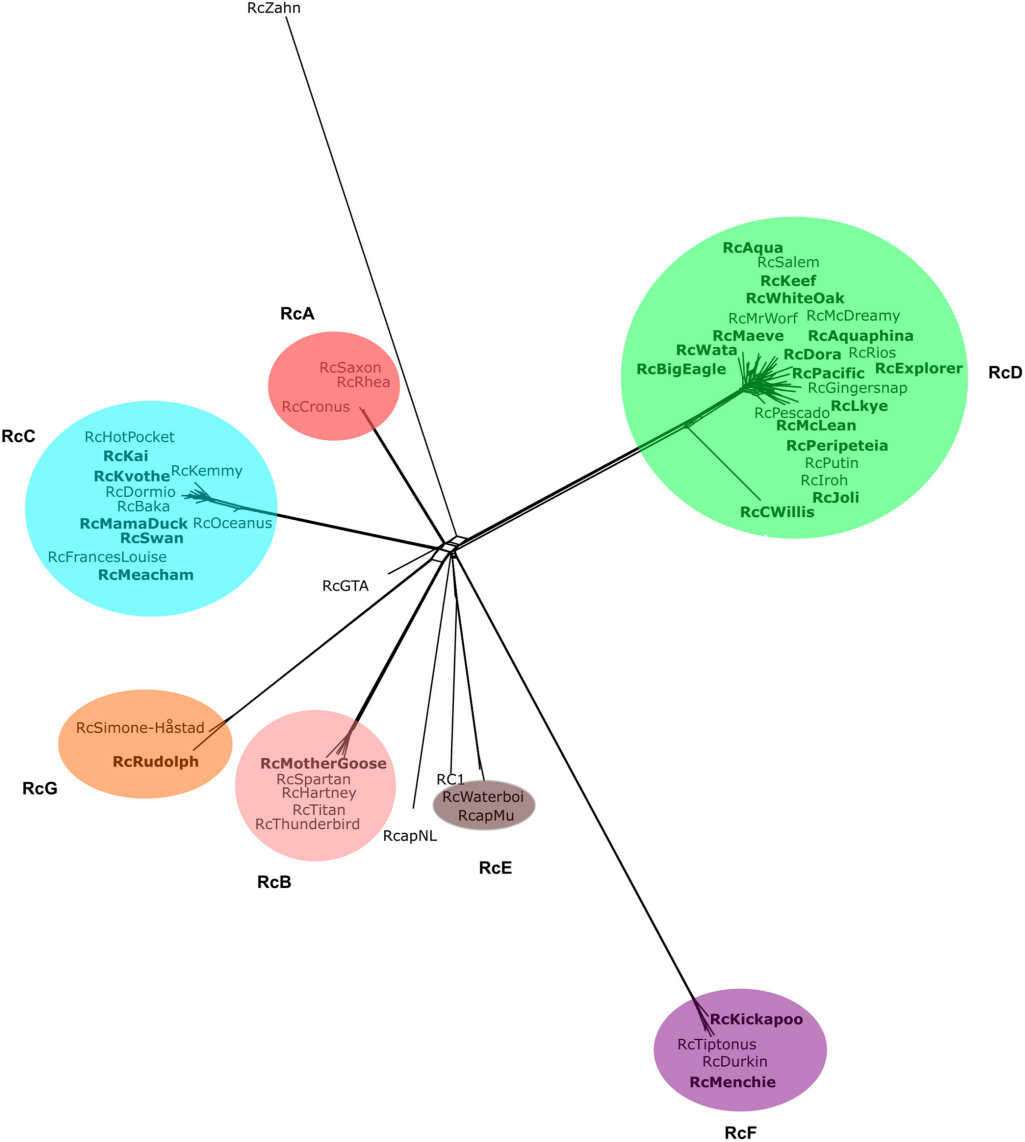
Network phylogeny of R. capsulatus bacteriophages. The predicted proteins of all 53 sequenced R. capsulatus phages and those found in the 14,087-bp RcGTA structural gene region were sorted into 885 families (phams) according to shared amino acid sequence similarities using Phamerator (16). Each genome was then assigned values reflecting the presence or absence of members of each pham; the genomes were compared and displayed using SplitsTree (18). Clusters are indicated with colored ovals. Phage names in bold indicate phages added to this collection as part of this study.

All 24 phages share substantial similarity with previously reported isolates, with all sharing at least 77% average nucleotide identity (ANI) (17) with at least one other isolate (Table 1; Fig. 1). RcD phages continue to be the most represented, with all but one of the 15 assigned to cluster RcD and sharing >90% ANI with other RcD phages. RcCWillis shares only 77 to 79% ANI with any other RcD phage. Of its 97 genes, 19 are unique.

None of the five phages assigned to cluster RcC show such divergence. They share 98 to 99% ANI with 5 previously identified members, but RcKemmy continues to be an outlier, sharing 85 to 86% ANI with the 10 other members. RcMotherGoose is now the most divergent of the 5 known RcB phages, with 89 to 91% ANI to any other member and 4 unique genes. RcF phages RcKickapoo and RcMenchie cluster tightly with the other two members, sharing 98 to 99% ANI.

Last, RcRudolph forms the new cluster, RcG, with RcSimone-Håstad. The ANI between them is 82%. They share 72 genes, with 14 in RcRudolph and 8 in RcSimone-Håstad being unique. RcSimone-Håstad previously stood out as a singleton isolated outside the United States. The discovery of RcRudolph from an Illinois pond demonstrates that this type is not unique to Sweden.

### Data availability.

Sequence reads have been deposited at NCBI under BioProject accession number PRJNA865772 and SRA accession numbers SRX17116950 to SRX17116973 (Table 1).
